# Evaluating the Accuracy of Imputation Methods in a Five-Way Admixed Population

**DOI:** 10.3389/fgene.2019.00034

**Published:** 2019-02-05

**Authors:** Haiko Schurz, Stephanie J. Müller, Paul David van Helden, Gerard Tromp, Eileen G. Hoal, Craig J. Kinnear, Marlo Möller

**Affiliations:** ^1^DST-NRF Centre of Excellence for Biomedical Tuberculosis Research, South African Medical Research Council Centre for Tuberculosis Research, Division of Molecular Biology and Human Genetics, Faculty of Medicine and Health Sciences, Stellenbosch University, Cape Town, South Africa; ^2^South African Tuberculosis Bioinformatics Initiative (SATBBI), Faculty of Medicine and Health Sciences, Stellenbosch University, Cape Town, South Africa

**Keywords:** imputation, accuracy, quality, admixture, 1000 Genomes, African, CAAPA, AGR

## Abstract

Genotype imputation is a powerful tool for increasing statistical power in an association analysis. Meta-analysis of multiple study datasets also requires a substantial overlap of SNPs for a successful association analysis, which can be achieved by imputation. Quality of imputed datasets is largely dependent on the software used, as well as the reference populations chosen. The accuracy of imputation of available reference populations has not been tested for the five-way admixed South African Colored (SAC) population. In this study, imputation results obtained using three freely-accessible methods were evaluated for accuracy and quality. We show that the African Genome Resource is the best reference panel for imputation of missing genotypes in samples from the SAC population, implemented via the freely accessible Sanger Imputation Server.

## Introduction

Over the past decade, genotyping technologies for genome-wide association studies (GWAS) have allowed for extensive and rapid genotyping of common variants (Ding and Jin, 2009; Ragoussis, 2009; Vergara et al., 2018). Commercial single nucleotide polymorphism (SNP) genotyping arrays contain between 300 000 and 2.5 million markers, but none have complete coverage of the human genome. Genotype imputation can be used to improve both coverage and power of a GWAS by inferring the alleles of un-genotyped SNPs based on the linkage disequilibrium (LD) patterns derived from directly genotyped markers and comparing them to a suitable reference population (Marchini and Howie, 2010; Pei et al., 2010; Malhotra et al., 2014). These imputed variants can then be used for association testing, to improve fine-mapping of a target region, or to conduct a meta-analysis.

Meta-analysis is a powerful and commonly used technique, but if the study data were generated using different platforms, there may be a reduction in statistical power due to minimal overlap between the genotyped markers. To overcome this reduction in power, imputation may be used to increase the marker overlap between datasets, thereby improving the power of a meta-analysis (Anderson et al., 2008; Marchini and Howie, 2010; Hancock et al., 2012; McRae, 2017).

Imputation is dependent on the adequate matching of haplotypes based on LD and thus it is essential that the reference population is genetically similar to the population being imputed. Numerous reference datasets are freely available online and can be used for imputation via suitable imputation software. These include amongst others, the 1000 Genomes phase 3 data (1000G) (Sudmant et al., 2015), the Human Genome Diversity Project (Cavalli-Sforza, 2005), Haplotype Reference Consortium (HRC) (McCarthy et al., 2016) and the HapMap consortium (International HapMap 3 Consortium et al., 2010). Most of the above-mentioned reference panels focussed mainly on representing the European population and data for African populations and admixed populations containing African ancestry is limited.

African and admixed populations are more heterogeneous in their haplotype block structure and, as such, would benefit from a larger reference dataset incorporating more genetic diversity (Vergara et al., 2018). Reference datasets of this nature would increase the chances that an observed haplotype is present in the reference data, thereby greatly improving the imputation accuracy for African and admixed individuals with African ancestry. Fortunately, recent years have seen a substantial increase in the representation of African populations in the 1000G data (Sudmant et al., 2015) and additional databases focusing on representing African populations have been established. The Consortium on Asthma among African ancestry populations in the Americas [CAAPA, (Mathias et al., 2016)] reference panel is available for download from dbGap with Accession ID:phs001123.v1.p1 (access required) and the African Genome variation project (AGVP) (Gurdasani et al., 2015) as well as the African Genome Resource^1^ (AGR, not publicly available) are three resources which have recently become a viable option for accurate imputation of African populations.

The AGR^1^ contains the largest collection of haplotypes of African origin, with all the 1000G samples and an additional 2000 samples from Uganda, 100 samples from each of a set of five populations from Ethiopia, Egypt, Namibia (Nama/Khoesan), and South Africa (Zulu). The AGR contains 97 004 203 biallelelic SNPs spanning the autosomes and the X chromosome for 4 956 samples^1^. The 1000G reference panel contains 84 237 642 biallelic SNPs for 2 504 samples selected from 26 populations across Europe, Asia, the Americas, South-, and East-Asia (Sudmant et al., 2015). The CAAPA reference panel contains whole-genome sequences for 883 samples recruited into 19 case-control studies on asthma in the Americas. A total of 31 163 897 autosomal SNPs are included on the panel for imputation (Mathias et al., 2016).

Apart from choice of reference panel, the software used also affects the imputation accuracy (Hancock et al., 2012). Many imputation software packages are freely available and have been previously tested and validated for accuracy, including Impute2 (Howie et al., 2009), Beagle (Verma et al., 2014), MaCH, MaCH-Minimac and MaCH-Admix (Roshyara et al., 2016). These imputation software packages were evaluated in African and African-American populations using different reference panels and produced varying degrees of imputation quality and accuracy (Hancock et al., 2012; Roshyara et al., 2016).

Huang et al. (2009) tested imputation accuracy in 29 populations using the HapMap reference and showed that the highest imputation accuracy was achieved for European populations, followed by East-Asian, Central- and South-Asian, American, Oceanian, Middle-Eastern, and African populations. An additional finding from this study was that combining multiple reference populations resulted in improved imputation accuracy for any population analysed (Huang et al., 2009). While more appropriate reference panels are now available, which would increase the accuracy of imputation in African individuals, these results indicate that there are difficulties when imputing populations for which there is a limited number of reference individuals.

Imputation accuracy has previously been assessed for African populations (Huang et al., 2009; Hancock et al., 2012; Roshyara et al., 2016) and for populations with two- or three-way admixture, with results reaching over 75% accuracy (Nelson et al., 2016). In the present study, we assessed the accuracy of imputation in the five-way admixed South African Colored (SAC) population. The SAC population contains genetic contributions from Bantu-speaking Africans, KhoeSan, Europeans, and South- and East-Asians (de Wit et al., 2010; Daya et al., 2013). While, imputation in this population has been conducted previously and the resulting data used for association analyses (Chimusa et al., 2014), the accuracy of imputation in this highly admixed population is yet to be evaluated.

Here we assessed the quality and accuracy of results obtained from imputation in the SAC population and show that the AGR reference panel - accessed via the Sanger Imputation Server- produced the highest quality and accuracy in imputed data. An in-house protocol using IMPUTE2 and 1000G reference panel imputed more variants than Sanger (AGR) but at a slightly reduced quality and accuracy.

## Methods

### SAC Data

Two sources of data for the SAC cohort were available, namely genotypes obtained using the Affymetrix 500k array containing 500 000 SNP markers (Affymetrix, California, United States) and the Illumina (Illumina, California, United States) multi-ethnic genotyping array (MEGA) with 1.7 million markers. This study was carried out in accordance with the recommendations of the Health Research Ethics Committee of Stellenbosch University (project registration number S17/01/013, S17/02/037, and 95/072) before participant recruitment and written informed consent was obtained from all study participants prior to blood collection. All subjects gave informed consent in accordance with the Declaration of Helsinki. The protocol was approved by the Health Research Ethics Committee of Stellenbosch University.

Genotype data obtained using the Affymetrix and MEGA arrays were subjected to iterative quality control (QC) using PLINK v1.9 (Purcell et al., 2007; Chang et al., 2015) as previously described (Schurz et al., 2018), with the exception of related individuals not being removed. Individuals missing more than 10% genotype information and SNPs with more than 2% missingness were removed, as well as any variants with a minor allele frequency (MAF) below 5% as well as loci with excessive heterozygosity (a detailed description of the filtering process can be found in Supplementary Data S3). All remaining missingness in the data is randomly distributed (data not shown) and the stringent SNP filter was used to ensure there are no incorrectly genotyped variants in the data that could influence the imputation accuracy (Supplementary Data S4).

These QC steps were iterated until no additional variants or individuals were removed, and concluded with a sex-concordance check to remove individuals with incorrect sex information. Genotype Harmoniser version 1.4.15 (Deelen et al., 2014) was used to strand align the two datasets to the 1000 Genomes Phase 3 reference panel [human genome build 37, (Sudmant et al., 2015)], update SNP IDs and remove any variants not in the reference panel. For the strand alignment a minimum LD value of 0.3 with at least three flanking variants was required for alignment. A secondary MAF alignment was also used at a threshold of 5%. Finally, the minimum posterior probability to call genotypes in the input data was left at the default value of 0.4.

### Phasing and Imputation

Three different reference panels were used to conduct five protocols of phasing and imputation in order to assess which performed best for our admixed population (Table 1). The first protocol was an in-house method where the Affymetrix data (PLINK files) were phased using SHAPEIT v2 (Delaneau et al., 2012), using the default effective population size of 15 000. Imputation was then performed using IMPUTE2 v2.3.2 (Howie et al., 2009) and the 1000G Phase 3 reference panel (Sudmant et al., 2015), with default parameters except for the effective population size, which was set to 15 000 for consistency with the haplotype phasing process.

**Table 1:** Haplotype phasing and genotype imputation methods used.

Protocol number	Server	Reference Panel	Phasing software	Imputation software
1	In-house	1000G	ShapeITv2	IMPUTE2
2	Sanger Imputation Server	1000G	ShapeITv2	PBWT
3	Sanger Imputation Server	AGR^1^	ShapeITv2	PBWT
4	Michigan Imputation Server	1000G	ShapeITv2	Minimac3
5	Michigan Imputation Server	CAAPA^2^	ShapeITv2	Minimac3


*^1^AGR, African Genome Resource. ^*2*^CAAPA, Consortium on Asthma among African-ancestry Populations in the Americas*.

The second-, and third protocol made use of the Sanger Imputation server^1^ (SIS). Genotypes from the Affymetrix 500k array in PLINK file format were converted to Variant Call Format (VCF) using PLINK v1.9 and then uploaded to the server where phasing was performed using SHAPEITv2.r790 (Delaneau et al., 2012) followed by imputation using the Positional Burrows-Wheeler Transformation (PBWT) algorithm (Durbin, 2014). Imputation was performed in two separate runs: the first run made use of the 1000G Phase 3 reference panel for imputation, and the second run made use of the African Genome Resource panel.

The fourth- and fifth protocol made use of the Michigan Imputation server [MIS, (Das et al., 2016)]. PLINK files were converted to VCF using PLINK v1.9 and uploaded to the server for two imputation runs, both of which were run on the QC and imputation mode. SHAPEITv2.r790 was used for haplotype phasing in both runs followed by imputation using the Minimac3 algorithm (Das et al., 2016). For the first run the mixed population option was used for the QC and haplotype phasing was performed followed by imputation with the 1000G Phase 3 reference panel. For the second imputation run, it was mandatory for the African-American population to be selected for QC when imputing with the CAAPA reference panel.

In summary, all of these methods implement a Hidden Markov Model (HMM) in different ways. Impute2 uses the Markov-chain to implement the HMM, while minimac3 uses a Monte-Carlo procedure to implement HMM (Li et al., 2010). PBWT also works on a Monte Carlo iteration but instead of HMM it infers haplotypes using a Positional Burrows Wheeler Transformation. All these imputation algorithms do a number of iterations of phasing (haplotype inference) and imputation and then the probabilities for each genotype are averaged for all iterations to give the posterior probability for each imputed genotype (Supplementary Data S2).

Although haplotype pre-phasing has been shown to decrease imputation accuracy slightly it was used in this study for consistency between the protocols (the Michigan server did not have an option to not phase data) and to increase the speed of imputation (Howie et al., 2009).

For all imputation runs, the reference panels included all available populations since using an all-inclusive reference panel is known to improve imputation accuracy (Huang et al., 2009). Of the five variations of imputation performed, only the MIS (CAAPA) run was incapable of performing imputation on the X chromosome. Results for the X chromosome have, however, been included for the other four imputation runs since the accuracy of X-linked imputation has not been previously evaluated.

### QC of Imputed Data

Imputed data were returned from the imputation software in one of two formats: either in the form of a VCF file, or in Impute2 (gen/sample) format and based on the format, one of two QC procedures was employed to convert the imputed data from genotype probabilities to actual genotypes. Data output from the two procedures were compared and showed complete overlap and can thus be used interchangeably.

#### Procedure 1

For the in-house imputation performed using Impute2, a gen/sample output file was obtained and converted to a PLINK file using GTOOL^2^ version 0.7.5. R version 3.2.4 was used to identify INDELS, which were removed using GTOOL (R Development Core Team, 2013). This was performed in order to more accurately assign SNP IDs and allele information when genotypes were called using GTOOL. The genotype calling threshold was set to 0.7, which was determined to have the best ratio of imputation accuracy and number of imputed variants (Supplementary Figure S1). Once genotypes were called, the resulting ped/map PLINK files were converted to bed/bim/fam PLINK files and all variants with no-call alleles were removed.

#### Procedure 2

For the imputation completed using the two online servers, VCF files were returned. The VCF files were converted to PLINK ped/map files using a genotype calling threshold of 0.7 (PLINK command: – vcf-min-gp command) and coding all no-call alleles as N (PLINK command: – output-missing-genotype N). INDELS and SNPs with no-call alleles were removed and the files were converted to PLINK bed format (bed/bim/fam).

### Imputation Quality and Accuracy

To assess imputation quality we considered the internal quality metrics obtained from each imputation protocol: the INFO score (in the case of IMPUTE2) and the r-squared value (for PBWT and Minimac3). Although, the info score and r-squared quality metrics are not directly comparable, they have shown to be highly correlated in two notable studies: one by Marchini and Howie (Marchini and Howie, 2010), and another by Browning and Browning (Browning and Browning, 2016). Both papers reported that the quality scores returned by several commonly used imputation software, including those utilized in the protocols of this study, are highly correlated. These values range from 0 to 1, where a higher value indicates increased quality of an imputed SNP. These quality metrics were used to assess within data quality, not between data quality. Median quality scores were plotted against MAF in order to determine how quality was affected by MAF and to assess which imputation protocol had returned the best quality data at a given MAF.

Imputation accuracy was assessed by extracting the overlapping individuals from the MEGA and imputed Affymetrix data and using PLINK, any variants that overlapped between the two platforms prior to imputation were removed. Between the two arrays there were only 41 815 variants genotyped on both platforms and they were evenly distributed across the genome and should not affect the analysis if removed post-imputation. The analysis was performed per chromosome and for each SNP the alleles were compared between the imputed Affymetrix data and the MEGA data. If both alleles of a SNP matched it would be considered a complete match (or a flip match if alleles were correct but strand swopped). If only one allele matched it was considered a half match and if no alleles matched it was considered a no-match. For each chromosome the total number of imputed variants was recorded and their distribution by MAF was plotted to determine how the number of variants correlated with MAF between the different imputation protocols.

To determine the imputation accuracy, the SNP overlap between the MEGA and imputed Affymetrix data was assessed. Within this overlap the number of SNPs that were complete-, flip-, half- or non-matched were recorded along with their average INFO score or r-squared value. Since SNPs that are flipped can be flipped to align a reference, or a different dataset if a meta-analysis is planned, the flipped SNPs were considered matches for the purposes of calculating imputation accuracy. Accuracy was calculated by comparing the proportion of SNPs in the overlap that were complete (or flipped) matches to the number of overlapping SNPs. This provided an indication of accuracy and error rate within the overlapping region and should be a good indication of overall imputation accuracy. These calculations were performed for the autosomes and the X chromosome separately in order to determine how accurately and with what quality the X-linked variants were imputed compared to the autosomal variants.

## Results

### Genotyping Data

After QC and strand alignment, 919 individuals and 239 612 variants with a genotyping rate of 99.39% remained in the Affymetrix 500k dataset, and 771 Individuals with 1 491 347 variants remained in the MEGA dataset with a genotyping rate of 99.43%. A total of 325 individuals were genotyped on both the Affymetrix and MEGA array and 43 140 SNP markers overlapped between the two platforms. Following imputation the 325 individuals with genotype data from both MEGA and Affymetrix were extracted from both the MEGA data and imputed Affymetrix data so that their imputed genotypes (Affymetrix) could be directly compared to their actual genotypes (MEGA) in order to determine genotyping accuracy. The 43 140 SNPs that were genotyped on both platforms were removed from both datasets after imputation in order to not skew the accuracy analysis.

### Imputation

For the SAC cohort, the best genotype imputation results obtained were from the in-house IMPUTE2 (with 1000G reference panel) and the Sanger imputation server (with the AGR reference panel) methods. The in-house method resulted in the most imputed variants across both the autosomes (60 438 387) and X chromosome (2 574 793), followed by SIS (AGR) (52 088 766 autosomal and 1 638 163 X-linked variants), while the SIS with 1000G reference panel had slightly fewer imputed variants than with the AGR panel (50 418 390 autosomal and 1 679 254 X-linked variants). The Michigan imputation server had only about half as many imputed variants as the other methods, for either reference panel (Table 2). The number of imputed variants that did not reach the genotype calling threshold (0.7) was lowest in the in-house method followed by the Michigan server results, and SIS (1000G) and SIS (AGR) had the highest percentage of variants not reaching genotype calling threshold (Table 2). When imputed Affymetrix variants were compared to the MEGA genotypes, the SIS (AGR) data had the highest accuracy (within the overlapping region) on both the autosomes (89.27%) and X chromosome (90.21%). The imputation accuracy for the in-house and SIS (1000G) method was very similar, with the in-house method having a slightly lower genome wide error rate. The accuracy of the Michigan server was good on the autosomes (∼62-83%) but lacking for the X chromosome (∼65%) (Table 3). The SIS (AGR) imputed the least X-linked variants, but at the highest accuracy, whereas the in-house method had twice as many X-linked variants as Sanger with only a 1.28% drop in accuracy (Tables 3, 4).

**Table 2:** Number of imputed variants and variants overlapping with MEGA as well as the percentage of calls that did not reach the genotype calling threshold (0.7). Imputed number of SNPs is given in millions and Overlapping number is given per ten thousand.

Method	Reference	Autosomes		X chromosome		% No calls
		Imputed^1^	Overlap^2^	Imputed^1^	Overlap^2^	
In-house	1000G	57.8	71.8	2.5	3.98	25.46
SIS	1000G	48.7	46.7	1.7	1.01	35.89
	AGR	50.5	60.6	1.6	1.43	44.18
MIS	1000G	28.6	47.8	1.3	2.79	35.22
	CAAPA	16.9	34.3	NA	NA	43.40


*^1^Number of SNPs in millions. ^*2*^Number of SNPs per ten thousand*.

**Table 3:** Genome wide error rate and accuracy of imputation on the autosomes and X chromosome.

Method	Reference	Accuracy in overlap (%)		GW Error rate in overlap (%)
		Autosomes	X chromosome	
In-house	1000G	88.00	87.93	11.98
SIS	1000G	87.15	88.12	12.83
SIS	AGR	89.27	90.21	10.70
MIS	1000G	83.68	69.89	17.084
MIS	CAAPA	62.39	NA	37.61


**Table 4:** Number of SNPs and accompanying median quality score for the three categories, within the MEGA overlapping region.

Method	Reference	Autosomes						X chromosome					
		Total		Half		No		Total		Half		No	
In-house	1000G	632^a^	0.78	38^a^	0.36	48^a^	0.89	35^a^	0.73	2.7^a^	0.37	2.1^a^	0.83
SIS^1^	1000G	407	0.79	25	0.46	35	0.87	8.9	0.8	0.5	0.56	0.7	0.88
	AGR	541	0.79	23	0.5	42	0.89	12.9	0.83	0.6	0.6	0.8	0.89
MIS^1^	1000G	400	0.69	45	0.11	33	0.83	19.5	0.57	7.1	0.08	1.3	0.70
	CAAPA	214	0.68	105	0.03	24	0.76	NA					


*^a^Number of SNPs in thousands*.

**FIGURE 1:**
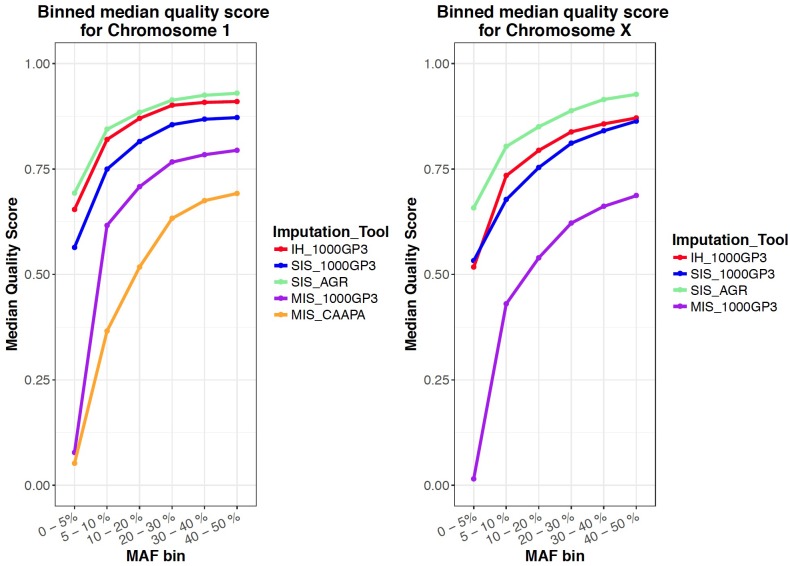
Mean quality score for all variants in a certain MAF range for all imputed datasets.

**FIGURE 2:**
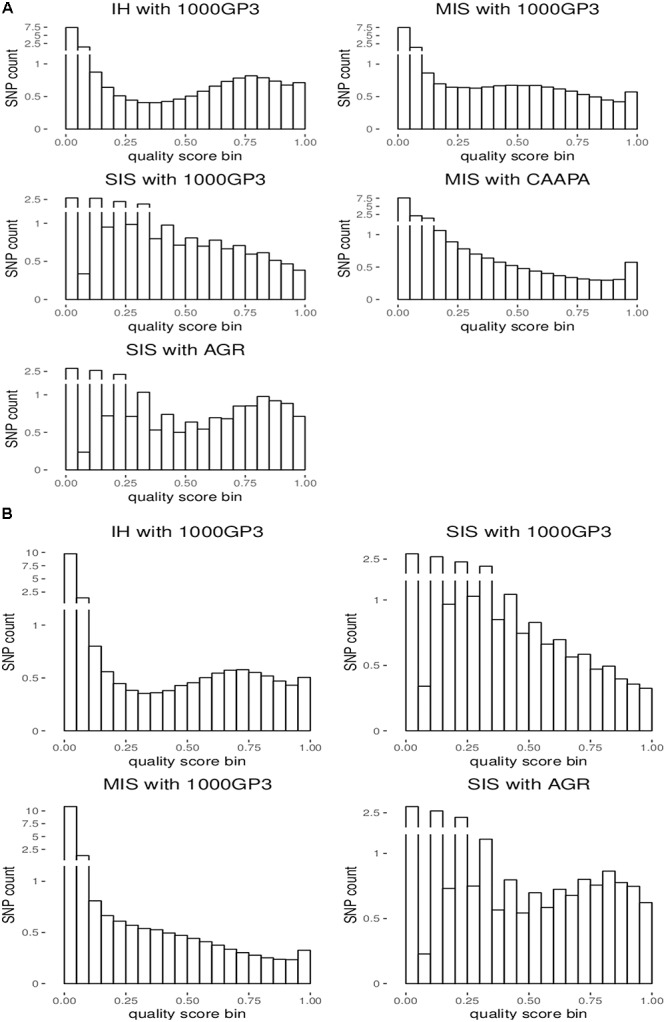
Distribution of the number of imputed SNPs by quality score for **(A)** chromosome 1 and **(B)** the X chromosome.

For the autosomes and X chromosome, the SIS (AGR) produced the best imputation quality across all MAF ranges, closely followed by the in-house method where quality was second to SIS (1000G) only for low MAF (0-1%) variants on the X chromosome (Figure 1). The Michigan server produced the lowest quality imputation according to internal quality metrics (Figure 1 and Table 4). The median quality score was comparable across all autosomal chromosomes and thus only chromosome 1 is shown as a representation of the autosomes and for comparison to the X chromosome (Figure 1). Figure 2 confirms that the SIS (AGR) method and the in-house method produced the best imputation quality since more SNPs were imputed at high quality for both Chromosome 1 and the X chromosome. Since the SIS (AGR) has the largest number of imputed genotypes not reaching the calling threshold, a trade-off between quality and number of variants exists between SIS (AGR) and the in-house method.

## Discussion

Imputation accuracy was previously evaluated in African and three-way admixed populations, but we have performed the first evaluation in a five-way admixed population. The imputation accuracy in African-American individuals (considered to be three-way admixed) ranges from 78% (Malhotra et al., 2014) to 89% (Howie et al., 2009). Bantu-speaking Southern African individuals have been imputed with an accuracy of about 95% and even African San individuals had an imputation accuracy of 89% (Huang et al., 2009). In the present study, the SIS (AGR) and the in-house imputation protocol had similar accuracies (89% and 88%, respectively, Table 2) compared to previous results from African and admixed populations. It should however, be noted, that the clear majority of non-matching variants were ambiguous (Imputed genotype A/T and MEGA genotype G/C, or vice versa) and the majority of half-matched variants were imputed as monomorphic (data not shown). These ambiguous variants were imputed at high quality (Table 3) and were not removed when filtering on quality score, but could be removed or aligned to a reference allele using appropriate software (such as Genotype Harmonizer). However, removal of these ambiguous variants is not mandatory. When analyzing a single dataset, the ambiguous variants of interest can be compared to a relevant reference genome and then flipped. This is especially useful when conducting a meta-analysis since these variants will then be comparable even though they originate from different datasets. If these ambiguous variants are considered to be correctly imputed, then the accuracy of imputation with the SIS (AGR) increases to 96% while the accuracy of the in-house imputation protocol increases to 94%. Accuracy and quality can be further improved by removing half-matching variants by applying a quality score and MAF filter.

Since four of the five protocols were capable of imputing X-linked variants, and since the quality and accuracy of X chromosome imputation has not been previously tested, we included it for this analysis. The X chromosome had only slightly lower or higher imputation quality for all imputation runs when compared to the autosomes, indicating that X chromosome imputation can be performed with confidence (Tables 2, 3). Although not specifically analysed here, the quality of imputation at low MAF should also be noted: the imputation quality for rare variants was unexpected as large reference panels with the correct populations are required to accurately impute rare variants (Kim et al., 2015; Zheng et al., 2015; Figure 1).

The biggest limitation for imputation in the five-way admixed population is the lack of a suitable reference panel. Imputation in the San population has been shown to have the lowest imputation accuracy (89%) compared to other African populations (Huang et al., 2009), which could be due to a lack of applicable reference individuals. Since the main ancestral component in the SAC population is KhoeSan (Daya et al., 2013) this could affect the accuracy and quality of imputation in this population. However, this has improved due to the addition of KhoeSan individuals in the AGR and 1000G reference panels.

In conclusion, we have shown that imputation of the SAC population is feasible and produces quality data on both the autosomes and X chromosome. While the SIS (AGR) imputation had the best quality and accuracy, the in-house protocol using Impute2 and 1000G Phase 3 also produced imputed data of a high standard and had the highest number of imputed variants. This protocol may prove especially useful in the case of a meta-analysis where one wishes to maximize SNP overlap between datasets. As the number of applicable reference populations and individuals grows, imputation accuracy will improve for African and admixed populations, but it remains the gold-standard to Sanger sequence a variant of interest to confirm that the imputed variant is present in the population prior to conducting further research.

## Data Availability

Summary statistics for the quality and accuracy assessment of the SAC data will be made available to researchers who meet the criteria for access to confidential data after application to the Health Research Ethics Committee of Stellenbosch University. Requests can be sent to: MM, E-mail: marlom@sun.ac.za.

## Author Contributions

HS, SM, GT, CK, and MM conceived the idea for this study. HS and SM performed the data QC. SM conducted phasing, imputation, and quality assessment. HS performed the accuracy assessment and wrote the first draft. All authors contributed to writing and proofreading for approval of the final manuscript.

## Conflict of Interest Statement

The authors declare that the research was conducted in the absence of any commercial or financial relationships that could be construed as a potential conflict of interest.

## Footnotes

1 https://imputation.sanger.ac.uk/

2 http://www.well.ox.ac.uk/$/sim$cfreeman/software/gwas/gtool.html

## Abbreviations

1000G: 1000 Genomes Phase 3 reference panel
AGR: African Genome Resource
AGVP: African Genome variation project
CAAPA: Consortium on Asthma among African ancestry populations in the Americas
HRC: Haplotype Reference Consortium
MEGA: Multi ethnic genotyping array
MIS: Michigan imputation server
PBWT: Positional Burrows-Wheeler Transformation
SAC: South African Colored
SIS: Sanger imputation server.
