# Supplementary material for: Evaluating the Accuracy of Imputation Methods in a Five-Way Admixed Population
Source: Front Genet. 2019 Feb 5;10:34. doi: 10.3389/fgene.2019.00034 (PMC6370942; doi:10.3389/fgene.2019.00034)
Supplement: Supplementary file 1 [file Table_1.DOCX]

Supplementary figure: S1


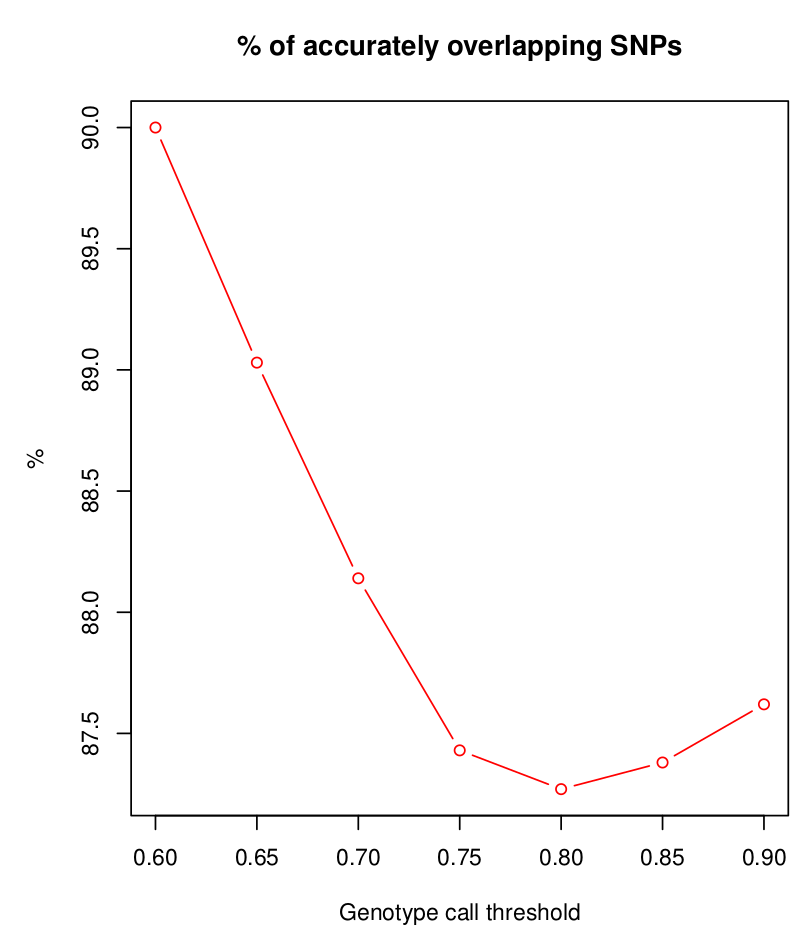


**Figure S1:** Percentage of overlapping variants that match between the imputed and MEGA data for different genotype calling thresholds.
