# Supplementary material for: Evaluating the Accuracy of Imputation Methods in a Five-Way Admixed Population
Source: Front Genet. 2019 Feb 5;10:34. doi: 10.3389/fgene.2019.00034 (PMC6370942; doi:10.3389/fgene.2019.00034)
Supplement: Supplementary file 2 [file Table_2.DOCX]

Supplementary data: S2

Impute2:

Imputation occurs by iterating through a two-step process of phasing typed SNPs and imputing untyped SNPs. First haplotypes are estimated based on sequence similarity of SNPs in both the study data and reference panel, thereby creating a custom reference panel for each study haplotype in each region of the genome. Once haplotypes have been estimated they are assumed to be correct and alleles are imputed into the study data (step 2). Each phasing and imputation step are driven by a Hidden Markov Model (HMM), which can be used to update an individual’s haplotype by constructing them as a set of imperfect mosaics of a set of template haplotypes. To account for phasing uncertainty, the two steps are iterated in a Markov Chain Monte Carlo (MCMC) framework. The MCMC algorithm runs for several iterations and then the probabilities from the imputation step are averaged across iterations to produce marginal posterior genotype probabilities at each untyped SNP.

Minimac3:

Minimac3 implements a Monte-Carlo Haplotyping procedure to estimate haplotypes in a sample of genotyped individuals. It assigns a random pair of haplotypes to each individual that is consistent with observed haplotypes. This involves randomly ordering alleles at each heterozygous site and sampling alleles at untyped sites according to the population frequencies. The haplotypes are updated for each individual by using the current set of haplotype estimates for all individuals as templates and sampling hypothetical mosaic states proportional to the likelihood. These mosaic haplotypes are estimated using a HMM which resolves a set of untyped genotypes into an imperfect mosaic of several template haplotypes. Inferences are then made about the sequence of mosaic states that best describe the observed genotype. Knowledge of these mosaic states will implicitly order alleles at heterozygous states and suggest an allele for each untyped location. This procedure is iterated so that new mosaic states are estimated, and haplotypes are updated accordingly to ensure they match the observed genotypes. After several rounds of refinement, a pair of consensus haplotypes are generated for each individual. The consensus haplotype pair is the pair that minimises total switch error when compared to the haplotypes sampled in each iteration.

PBWT: (Imputation methods for this has not been published)

PBWT assigns untyped alleles by aligning haplotypes based on sequence similarity using the Burrows–Wheeler transformation algorithm also used in DNA sequence alignment. First it builds a positional prefix array in which haplotypes are sorted in order of reversed prefixes order at a specific position, enabling for matches to be easily identified. This is achieved by sweeping through the data by position and building the next positional prefix array from the previous array. Haplotypes in the array are sorted by alleles at a specific position, ensuring that the sort order from the previous position is retained for all haplotypes with the same allele value. The next step is to build a divergence array. Sorting the haplotypes means that each haplotype in the prefix array will be adjacent to the haplotype with the longest match. The length of a match between any pair of non-adjacent haplotypes is the minimum of the length of the matches between all haplotypes occurring in between them, in sorted order. The divergence array records the maximal matches between each haplotype and its predecessor in sorted order. Next all matches are identified between haplotypes that are longer than a specific length. In the sorted array all haplotypes with this minimum length will be adjacent to each other, thereby forming a block. The algorithm iterates over all haplotypes in sorted order and collects all that have matches reaching the specified length. If a match shorter than the specified length is identified it indicates a break between blocks and instead reports matches from the previous block. After matches of certain length have been identified the set with maximal match length is identified and reported. At each position the algorithm iterates through the sorted haplotypes looking for a maximal match for each haplotype. Finally these prefix arrays with maximal length matches can be compressed and used for imputation. The compression means that less storage space is used and the sort order and maximal length matches in the array speed up imputation. However the exact mechanisms of imputation are not clear and have not been published.
