# Supplementary material for: Evaluating the Accuracy of Imputation Methods in a Five-Way Admixed Population
Source: Front Genet. 2019 Feb 5;10:34. doi: 10.3389/fgene.2019.00034 (PMC6370942; doi:10.3389/fgene.2019.00034)
Supplement: Supplementary file 3 [file Table_3.DOCX]

Supplementary data: S3

At the time of genotyping, the Affymetrix 500k SNP array (for 500 000 common SNPs) developed by Illumina was the most appropriate array for genotyping of individuals of South African admixed ancestry as it contains an extensive collection of markers from populations of the African Diaspora. Following standard genotype calling procedures and quality control, the raw dataset consisted of 397 337 variants successfully genotyped for 947 samples; 102 663 SNPs were not successfully genotyped for this cohort.

The 1000GP reference panel was used in conjunction with dbSNP to update the base pair position and/or chromosome number for variants lacking this information. Of these variants, 90 were successfully updated and 291 were removed, leaving 397 046 variants. Genotype Harmonizer v 1.4.20 was used to strand‑align the variants to the human reference genome. A total of 40 881 variants were excluded during the alignment phase: 2 786 due to the study variant being found in the reference dataset, but the alleles not being comparable, and 38 095 variants being excluded due to there not being enough non ambiguous SNPs in LD to assess the strand based on LD. After this exclusion, no non‑biallelic SNPs were found, leaving 947 samples with 356 165 variants.

Genotype quality control removed 28 individuals:11 due to individual genotype missingness, 13 due to ambiguity in the “sex” assignment, and 4 due to excess heterozygosity in their genotypes. Furthermore, 116 553 variants were removed during the iterative process of filtering for 2% SNP missingness and 5% MAF, leaving a dataset consisting of 919 individuals and 239 612 variants that passed QC with a genotyping rate of 99.39 %. These samples and variants passing our quality control procedure were then used as input for the various imputation protocols used in this study. Details of the loss of samples and variants during the filtering are detailed below:

**Summary description of Loss:**

| **Loss due to:** | **No. of Samples** | **No. of Variants** |
| --- | --- | --- |
| **IN** | **947** | **397 337** |
| 10% individual genotype missingness (--mind) | - 11 | NA |
| Incorrect ‘sex’ assignment | - 13 | NA |
| excess heterozygosity (4 SDs from mean) | - 4 | NA |
| remove variants for which SNP ID/bp pos could not be updated | NA | - 291 |
| variants not aligned with Genotype Harmonizer | NA | - 40 881 |
| 2% SNP genotype missingness (--geno) |  | - 86 517 |
| 5% SNP MAF (--maf) |  | - 30 036 |
|  |  |  |
| **Total Loss** | **(-28)** | **(-157 725)** |
| **OUT** | **919** | **239 612** |

**Detailed description of Loss:**

| file name | action | no. of samples | no. of variants |
| --- | --- | --- | --- |
| **data.bed/bim/fam** |  | **947** | **397 337** |
|  | remove variants for which SNP ID/bp pos could not be updated | NA | -291 |
| dataCleaned.bed/bim/fam |  | 947 | 397 046 |
|  | excl variants not aligned with GH |  | -40 881 |
| results.bed/bim/fam |  | 947 | 356 165 |
| **qc1.bed/bim/fam** | **First iteration of mind/maf/geno filter** | **-11(10% mind)** | **-86 517 (2% geno) -29 862 (5% maf)** |
|  |  | **936** | **239 786** |
| qc2.bed/bim/fam | Second iteration of mind/maf/geno filter | -0 (10% mind) | -0 (2% geno) --0 (5% maf) |
|  |  | 936 | 239 786 |
| qc3.bed/bim/fam | sex check | -13 |  |
| qc4.bed/bim/fam |  | 923 | 239 786 |
|  |  | **-0 (10% mind)** | **-0 (2% geno) -119 (5% maf)** |
| qc5.bed/bim/fam |  | **923** | **239 667** |
|  | excess heterozygosity (4 SDs from mean) | -4 | NA |
| passedhettest.bed/bim/fam |  | 919 | 239 667 |
| qc6.bed/bim/fam |  | **-0 (10% mind)** | **-0 (2% geno) -55 (5% maf)** |
|  |  | **919** | **239 612** |
|  | Second iteration of mind/maf/geno filter | -0 (10% mind) | -0 (2% geno) --0 (5% maf) |
| qc7.bed/bim/fam |  | 919 | 239 612 |
