# Supplementary material for: Evaluating the Accuracy of Imputation Methods in a Five-Way Admixed Population
Source: Front Genet. 2019 Feb 5;10:34. doi: 10.3389/fgene.2019.00034 (PMC6370942; doi:10.3389/fgene.2019.00034)
Supplement: Supplementary file 4 [file Table_4.DOCX]

Supplementary data: S4

**Table S4:** Proportion of sample-wise and SNP-wise missingness as reduced through the filtering procedure

|  | **Proportion of Sample‑wise missingness** | **Proportion of SNP‑wise missingness** |
| --- | --- | --- |
| **Directly after genotyping:** 947 / 947 samples, 397 337 / 500 000 SNPs | 0% | 20.533% |
| **QC 1:** 936 / 947 samples, 239 786 / 397 337 SNPs | 1.16% | 32.68% |
| **QC 5:** 923 / 936 samples, 239 667/ 239 786 SNPs | 1.39% | 0.049% |
| **QC 6:** 919 / 923 samples, 239 612 / 239 667 SNPs | 0.43% | 0.023% |
